# A method for in vivo treatment verification of IMRT and VMAT based on electronic portal imaging device

**DOI:** 10.1186/s13014-021-01953-9

**Published:** 2021-12-04

**Authors:** Jun Zhang, Xiuqing Li, Miaomiao Lu, Qilin Zhang, Xile Zhang, Ruijie Yang, Maria F. Chan, Junhai Wen

**Affiliations:** 1 Department of Biomedical Engineering, School of Life Science, Beijing Institute of Technology, Beijing, China; 2 Department of Engineering Physics, Tsinghua University, Beijing, China; 3 Department of Radiation Oncology, Peking University Third Hospital, Beijing, China; 4 Medical Physics Department, Memorial Sloan Kettering Cancer Center, New York, NY 10065 USA

**Keywords:** Radiotherapy, EPID, Quality assurance, In vivo verification

## Abstract

**Background:**

Intensity-modulated radiation therapy (IMRT) and volume-modulated arc therapy (VMAT) are rather complex treatment techniques and require patient-specific quality assurance procedures. Electronic portal imaging devices (EPID) are increasingly used in the verification of radiation therapy (RT). This work aims to develop a novel model to predict the EPID transmission image (TI) with fluence maps from the RT plan. The predicted TI is compared with the measured TI for in vivo treatment verification.

**Methods:**

The fluence map was extracted from the RT plan and corrections of penumbra, response, global field output, attenuation, and scatter were applied before the TI was calculated. The parameters used in the model were calculated separately for central axis and off-axis points using a series of EPID measurement data. Our model was evaluated using a CIRS thorax phantom and 20 clinical plans (10 IMRT and 10 VMAT) optimized for head and neck, breast, and rectum treatments.

**Results:**

Comparisons of the predicted and measured images were carried out using a global gamma analysis of 3%/2 mm (10% threshold) to validate the accuracy of the model. The gamma pass rates for IMRT and VMAT were greater than 97.2% and 94.5% at 3%/2 mm, respectively.

**Conclusion:**

We have developed an accurate and straightforward EPID-based quality assurance model that can potentially be used for in vivo treatment verification of the IMRT and VMAT delivery.

## Introduction

Radiotherapy is an effective method for tumor treatment. Intensity-modulated radiation therapy (IMRT) and volume-modulated arc therapy (VMAT) have become increasingly common in radiation therapy, as they can control the irradiation area more accurately and enable the target area to receive a higher and more conformal dose. However, IMRT and VMAT are also more complicated than traditional three-dimensional conformal therapy, and the high dose gradients typically associated with these treatments need to be stringently validated before delivery [1].

Electronic portal imaging devices (EPID) have been gradually introduced for quality assurance and dose verification due to their fast image acquisition, high resolution, good dose linear response, and long-term stability [2–4]. Dose verification with EPID is mainly divided into pre-treatment dose verification [5–7] and in vivo dose verification [3, 4, 8–16]. A detailed description of EPID dosimetry investigations was presented by van Elmpt et al. [17]. Margalit et al. [18] observed that even with pre-treatment verification, unexpected errors occurred during patient treatment. Therefore, there is a strong need for in vivo patient-specific quality assurance procedure (QA).

In vivo verification using EPID can be carried out by using either the backward approach [3, 10, 19] or the forward approach [8, 11, 14, 20]. In the forward approach, van Elmpt et al. [8] used acquired open portal images to predict TI during treatment. This method requires repeated delivery of the plan to acquire the open image, which is time-consuming and may not detect some machine errors. Fuangrod et al. [21] and Woodruff et al. [20] used the method of Chythk et al. [11, 14] to calculate the TI from the fluence map; they used the Monte Carlo (MC) method to establish the scatter model, which needs to simulate the Linac, and the modeling process is complex.

Regardless of whether the backward approach or forward approach is used, it is necessary to establish the scatter model of EPID (including Linac scatter, patient scatter, and EPID internal scatter). In previous studies, two principal methods have been used to calculate the scatter kernel: simulation by the MC method [14, 22–24] and calculation by the analytical method based on measured data [9, 25]. The MC method requires a detailed EPID structure for modeling. Generally, the commercial EPID structure is not easy to obtain, so it is difficult to model and slow to calculate. In the analytical method, the commonly used data are the measurement data of the central axis, so the calculated data are the scatter kernel of the central axis, which is typically used to approximate the off-axis scatter fluence and does not reflect the actual response of the EPID. Moreover, when using the convolution/deconvolution method for calculation, it is performed in the spatial frequency domain using the fast Fourier transform algorithm [26, 27]. In this case, the scatter kernel is required to be spatially invariant, that is, the central axis scatter kernel is used at each point of the EPID plane. Li et al. [28] demonstrated that different regions should use different kernels, and using the same kernel results in discrepancies between computed and measured images.

Efforts to improve error detection sensitivity are ongoing, Passarge et al. [29] utilized cine EPID images to create a Swiss cheese error detection method to detect relevant dose errors and indicate the origin of the error. Alves et al. [30] also utilized cine EPID images to identify aperture errors and quantify the detection power of these real-time detection modules.

This study aims to propose a novel EPID model-based method for in vivo treatment verification. This model can accurately predict the TI from the fluence map extracted from the radiation therapy (RT) plan, and then the predicted TI is compared to the measured portal image. The parameters used in the model are analytically calculated using EPID measured data; the primary ray, the scatter ray, and the distribution of scatter values at different off-axis points are modeled and calculated separately. Compared with the method using only central axis measurement data, our method improves the calculation accuracy of off-axis points and simplifies the calculation process compared with the MC method.

## Materials and methods

### Equipment

Measurements were performed with a Varian Trilogy Linac (Varian Medical Systems, Palo Alto, CA) equipped with a Millennium 120 multileaf collimators (MLC). The EPID detector (Varian aS1000 flat panel detector) was positioned at 150 cm source to detector distance, covering a field size of 40 × 30 cm^2^ with a resolution of 1024 × 768 pixels. Dark and flood fields were acquired before the experiment. All measurements were performed using 6 MV X-rays and the acquisition software IAS3 (Image Acquisition System 3) and then processed with MATLAB (Math Work, Natick, MA). EPID images were captured using the integrated mode and the backscatter influence of the EPID support arm was removed [7].

### Prediction model

**Fig. 1:**
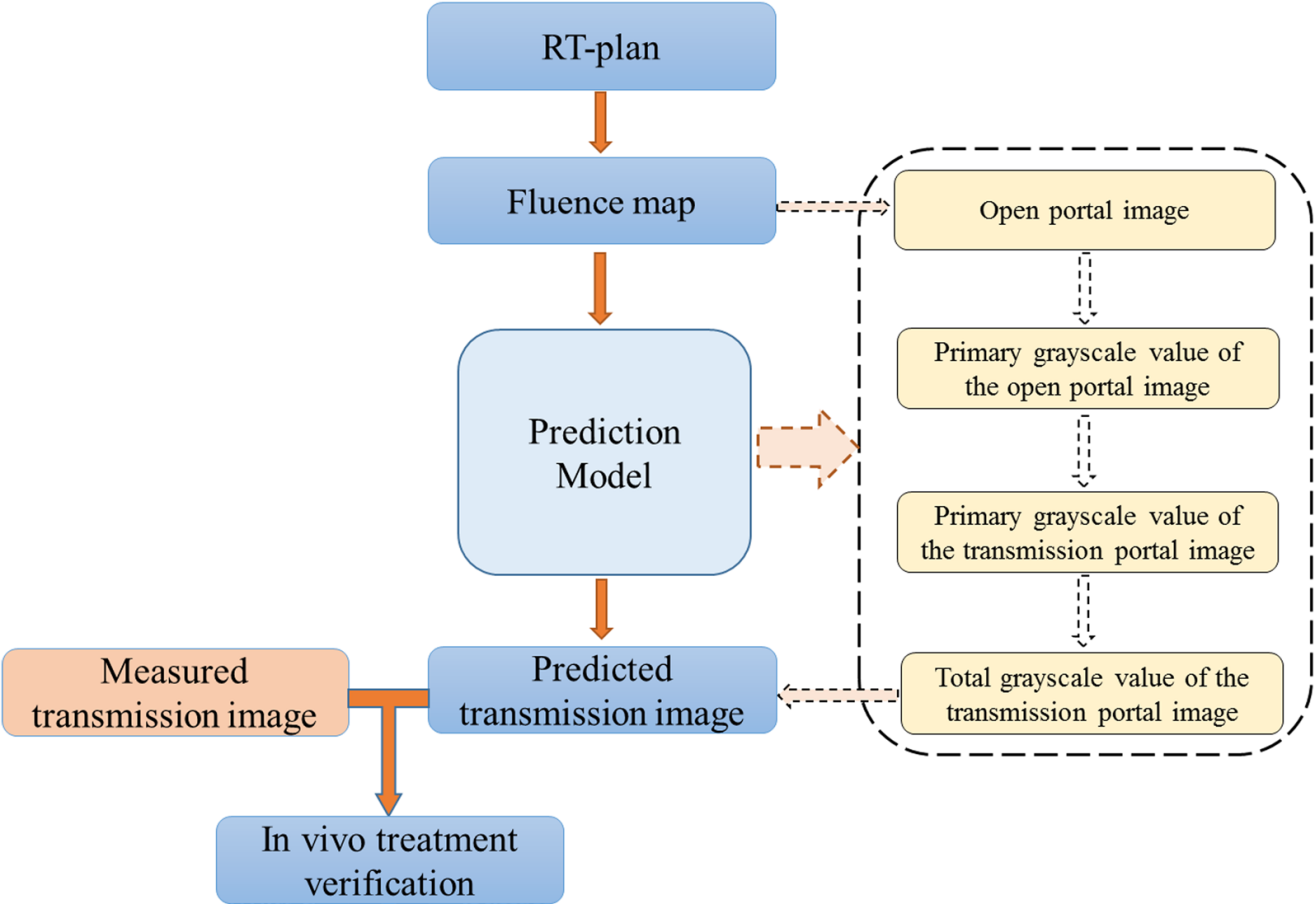
The flowchart of our model

The fluence map at SDD = 150 cm of each control point is extracted from the RT plan. The control points in the RT plan are up-sampled by a factor of five to improve the calculation accuracy, the beam parameters (MLC leaf positions, gantry angles and monitor units) linearly interpolate between control points. After penumbra and response corrections, the fluence map is pixel-by-pixel converted into an open portal image (without the phantom or patient in the beam). The open portal image is then converted into the TI after attenuation and scatter corrections. The prediction image of each control point is superimposed to obtain the integrated prediction image of the field, and the calculated TI is compared with the measured TI for in vivo treatment verification. Figure 1. shows the flowchart of the model.

**Fig. 2:**
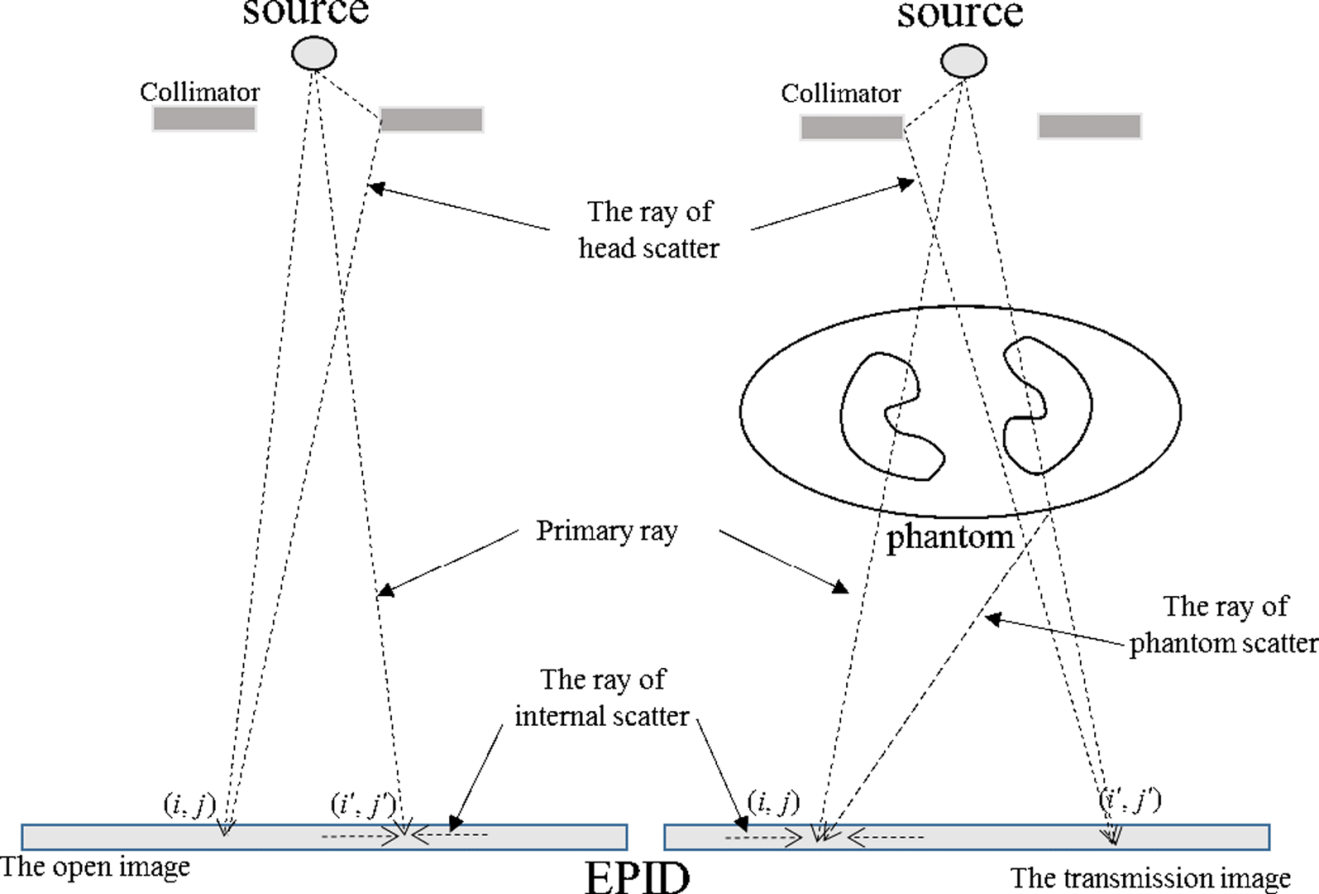
The progress of X-rays from the Linac to EPID

The total fluence of the EPID plane is composed of the primary fluence and the scatter fluence. X-rays emitted from the source and directly reaching the EPID plane after attenuation by the patient are called the primary fluence, and the ray generated after the interaction with the patient and Linac head or interaction within EPID are called the scatter fluence. In the EPID open portal image, the scatter fluence includes the scatter from the Linac head and the EPID internal scatter. In addition to these two types of scatter, the EPID TI also include the scatter fluence generated by the phantom or patient, as shown in Fig. 2. The primary ray follows the exponential attenuation law, and the scattered ray is related to the field size, the thickness of the phantom or patient, and the air gap. Therefore, the grayscale value of the primary ray in the calculated open image needs to be extracted first; after the phantom or patient attenuation correction, the primary value is added with the scatter value to obtain the predicted TI. The process of using the fluence map ($\psi$) to calculate the TI ($G_{tr}$) is expressed by the following formula:

1 $$G_{tr} = \frac{f(\psi \otimes K) \cdot HCM}{{GFO}} \cdot \exp \left( { - \frac{a \cdot t}{{1 + b \cdot t}}} \right) \cdot (1 + SPR)$$

where $f$ is the linear function of fluence to grayscale value; K is the convolution kernel, which is used to correct the theoretical fluence; Horn correction map (HCM) is used to match the horn effect on the fluence map; global field output factor (GFO) is used to calculate the primary value in the calculated open image; a and b are the attenuation coefficients; the scatter to primary ratio (SPR) of the EPID is used to calculate the scatter value in the transmission image.

The prediction model includes the following main steps:

1. Extract the fluence map of each control point from the RT plan and convert it into an open portal image;
2. Read the corresponding GFO according to the field size and off-axis distance to calculate the grayscale value of the primary ray in the calculated open image;
3. Calculate the primary grayscale value of the TI based on the equivalent thickness and attenuation coefficients;
4. Read the corresponding SPR according to the field size, equivalent thickness, off-axis distance, and exit gap of each point; then calculate the scatter grayscale value of each point in the EPID TI. The scatter value plus the primary value is the predicted TI.

#### Calculation of the open portal image from the fluence map

The theoretical fluence map extracted from the RT plan reflects the intensity values in the field and has the shape of 2D step functions with zero penumbra width. At field borders, penumbra effects occur due to the finite size of the focal source, scatter from collimator edges and transmission through rounded leaf ends. To model the influence of scattering inside the EPID, the theoretical fluence map is convolved with $k_{1}$ to correct the penumbra area. Furthermore, scatter was not perfectly identical for measured EPID and predicted EPID, and a $k_{2}$ was used to correct the penumbra response of the EPID [31, 32]. Finally, the fluence value is converted into the absolute EPID grayscale value through linear function. The flood field calibrations were utilized to normalize inherent pixel-to-pixel sensitivity variations to EPID [33], which causes a horn effect. The HCM is used to correct the fluence map to match the EPID image:

2 $$G_{0} = f(\psi \otimes k_{1} \otimes k_{2} ) \cdot HCM$$

3 $$k_{1} = c \cdot \exp \left( { - \frac{{r^{2} }}{{2\sigma^{2} }}} \right)$$

4 $$k_{2} = \sum\limits_{i = 1}^{3} {a_{i} \cdot \exp ( - b_{i} \cdot r)}$$

5 $$f(\psi ) = A \cdot \psi + B$$

where $G_{0}$ is the grayscale value of the open portal image and $c$, $\sigma$, $a_{i}$, $b_{i}$, $A$ and $B$ are fitting parameters; r is the off-axis distance ($r = \sqrt {i^{2} + j^{2} }$, $i$ and $j$ are the coordinate index values of each point in the EPID plane).

#### Calculation of the primary value in the open image

The grayscale value distribution in the open image, in addition to the primary value directly emitted by the Linac source, is also a scatter value generated by the interaction of the X-rays with the Linac head and EPID. The field output factor is used to calculate the primary value in the calculated open image. We define the global field output factor as the ratio of the total grayscale value to the primary grayscale value in the open image, and the GFO includes modeling the scatter value generated by the Linac head, and interaction within the EPID:

6 $$G_{0}^{p} (r) = \frac{{G_{0} (fs,r)}}{GFO(fs,r)}$$

where $G_{0}^{p} (r)$ is the primary grayscale value output by the Linac in the open image when the off-axis distance is $r$, $G_{0} (fs,r)$ is the total grayscale value in the open image, $GFO(fs,r)$ is the global field output factor when the field size is $fs$ and the off-axis distance is $r$.

Therefore, if the corresponding GFO value is known, the primary value output by the Linac can be calculated from the open portal image using Eq. (6).

#### Calculation of the primary value in the transmission image

EPID TI contains both primary and scatter components. The intensity of the primary ray decays as it passes through the phantom or patient, so the primary value extracted from the open portal image at each point can be modified according to exponential attenuations to obtain the primary value of the TI:

7 $$G_{tr}^{p} (t,r) = G_{0}^{p} (r) \cdot \exp \left( { - \frac{a(r) \cdot t}{{1 + b(r) \cdot t}}} \right)$$

where $G_{tr}^{p} (t,r)$ is the primary grayscale value in the TI when the thickness of the phantom or patient is $t$ and the off-axis distance is $r$.

The CT value of the phantom or patient is converted into the electron density relative to water by the relationship between the planning CT and electron density. The ray tracing algorithm [34] is then used to calculate the equivalent water thickness of the ray path.

#### Calculation of the total value in the transmission image

Since EPID TI contains the primary value and the scatter value, our model uses the SPR of EPID to calculate the scatter value in the TI.

The SPR of the EPID is defined as the ratio of the scatter value (including the scatter value from the Linac head, the phantom or patient, and the internal EPID) and the primary value corresponding to that point in the TI, as shown in Eq. (8):

8 $$SPR(L,fs,t,r) = \frac{{G_{tr}^{s} (L,fs,t,r)}}{{G_{tr}^{p} (t,r)}}$$

where $SPR(L,fs,t,r)$ represents the scatter to primary ratio of the TI; $G_{tr}^{S} (L,fs,t,r)$ is the scatter value of the TI when the air gap is L, the field size is $fs$, the thickness of the phantom or patient is $t$, and the off-axis distance is $r$.

The primary value that adds the scatter value is the total value of the TI, as shown in Eq. (9):

9 $$G_{tr}^{{}} (L,fs,t,r) = G_{tr}^{p} (t,r) \cdot (1 + SPR(L,fs,t,r))$$

### Calculation of the parameters

The model used a series of square fields (3–20 cm^2^) and thicknesses (0–40 cm) of solid water phantoms to calculate the parameters.

#### Calculation of the kernel, linear function and horn correction map

The fluence map in the RT plan is extracted, and the open portal image is acquired for four 100 MU square fields (sizes of 5, 10, 15, 20 cm). The parameters of the kernel function were derived from the calculated and measured open portal image.

A static field of 10 × 10 cm^2^ was delivered with a total number of monitor units ranging from 1 to 600 MU, and the fluence value and EPID grayscale value at the isocenter were extracted to calculate the linear conversion function. Different fields are corrected by the field output factors[31].

An open portal image of a 20 × 20 cm^2^ field is acquired, the image is normalized to the central axis value, and the value in the diagonal direction is the HCM.

#### Calculation of the global field output factor

As the portal image includes the primary value and the scatter value, the primary value at each point is independent of field size. The scatter value becomes greater as the field increases, so when the field size is infinitely small, the scatter value tends to zero; at this point, the EPID response is the primary value. Therefore, by measuring portal images of different field sizes, the least-squares method can be used to fit the EPID response value when the field size is zero, which is the primary value at each point, as shown in Eq. (10):

10 $$G^{p} (fs,t,r) = \mathop {\lim }\limits_{fs \to 0} G(fs,t,r)$$

where $G^{p} (fs,t,r)$ is the primary value in the portal image when the field size is $fs$, the thickness is $t$, the off-axis distance is $r$, and $G(fs,t,r)$ is the total value in the portal image.

In open portal images, the thickness of phantom $t = 0$. Therefore, the GFO in the open image can be calculated by Eq. (11). To calculate the primary value when the off-axis distance is $r$.

11 $$GFO(fs,r) = \frac{{G_{0} (fs,r)}}{{\mathop {\lim }\limits_{fs \to 0} G_{0} (fs,r)}}$$

#### Calculation of the attenuation coefficients

The primary ray follows the law of exponential decay during the penetration of the phantom. With increasing phantom thickness and off-axis distance, X-rays will undergo a hardening effect and a softening effect, so the attenuation coefficients of the central axis and off-axis are different [35], and the attenuation coefficient of each point needs to be calculated separately. We acquired EPID images of different field sizes and off-axis with different thicknesses (0–40 cm). Equation (10) is used to calculate the primary value at different off-axis points (0–10 cm) in the EPID plane at various thicknesses, and Eq. (12) is used to calculate the primary ray transmission at each point:

12 $$T_{{}}^{primary} (t,r) = \frac{{G_{tr}^{p} (t,r)}}{{G_{0}^{p} (r)}}$$

where $T_{{}}^{primary} (t,r)$ is the transmission of the primary X-rays when the thickness is $t$ and the off-axis distance is $r$.

The transmission of the primary ray follows the law of exponential attenuation, as shown in Eq. (13). The transmission of different solid water thicknesses and different off-axis distances calculated by Eq. (12) can be used to obtain the attenuation coefficient of solid water relative to each off-axis point.

13 $$T_{{}}^{primary} (t) = \exp \left( { - \frac{a(r) \cdot t}{{1 + b(r) \cdot t}}} \right)$$

#### Calculation of the scatter to primary ratio

The EPID TI includes the primary value and the scatter value. We can use Eq. (14) to calculate the SPR in the TI when the phantom thickness is $t$:

14 $$\begin{aligned} SPR(L,fs,t,r) &= \frac{{G_{tr}^{s} (L,fs,t,r)}}{{G_{tr}^{p} (t,r)}} \hfill \\ & = \frac{{G_{tr} (L,fs,t,r) - \mathop {\lim }\limits_{fs \to 0} G_{tr} (L,fs,t,r)}}{{\mathop {\lim }\limits_{fs \to 0} G_{tr} (L,fs,t,r)}} \hfill \\ \end{aligned}$$

**Fig. 3:**
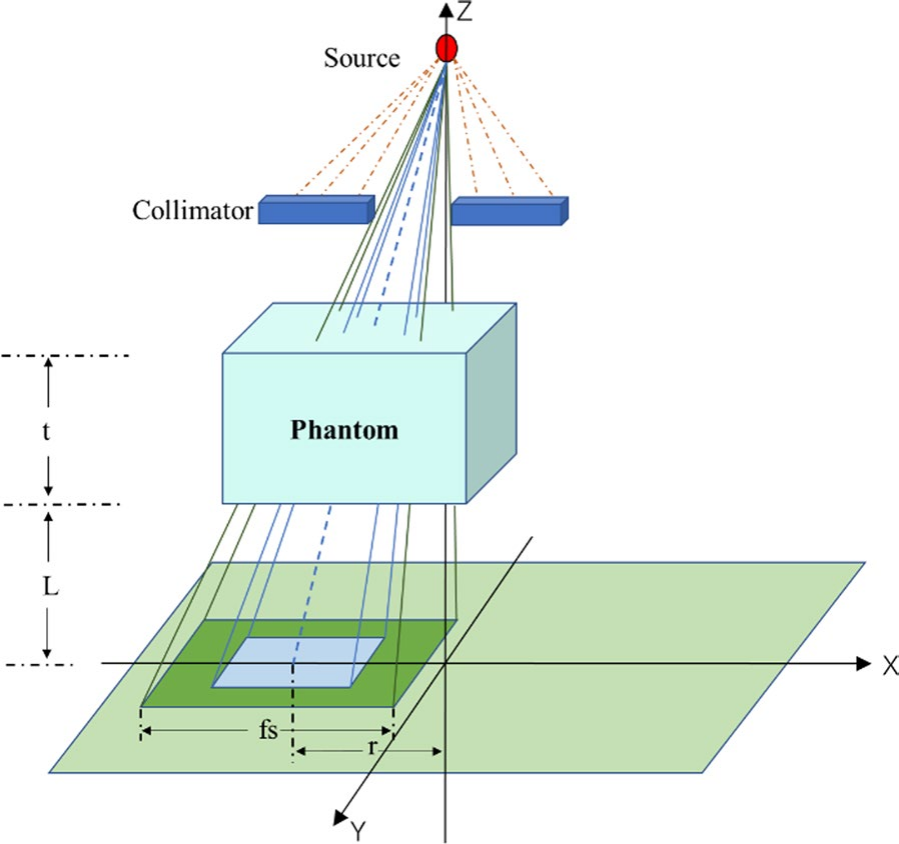
Measurement of the SPR when the thickness of the phantom was t, the exit gap was L, and the off-axis was r

The field size (fs), phantom thickness (t), off-axis distance (r), and air gaps (L) between the exit point and the EPID used to measure the SPR are shown in Table 1. The phantom used is solid water. When measuring the SPR of off-axis points, moving the MLC, a series of fields at each off-axis was acquired, as shown in Fig. 3.

**Table 1:** Data used to measure the SPR

fs (cm^2^)	t (cm)	r (cm)	L (cm)
3 × 3, 4 × 4, 5 × 5, 8 × 8, 10 × 10, 12 × 12, 15 × 15, 18 × 18, 20 × 20	0, 3, 5, 8, 10, 12, 15, 18, 20, 25, 30, 35, 40	0, 1, 2, 3, 4, 5, 6, 7, 8, 9, 10	20, 25, 30, 35, 40, 45, 50

A database of GFO, attenuation coefficients and scatter to primary ratios were established and the parameters assuming radial symmetry. For the same Linac and EPID, the measurement of these parameters needs to be performed only once. These parameter values can be obtained by linear interpolation according to the corresponding field size, equivalent thickness, off-axis distance, and exit gap corresponding to each point in the prediction model. When the field is irregular, the irregular field is converted into an equivalent square field [31].

### Validation

Two kinds of phantoms were used to verify the accuracy of the algorithm: a 40 × 40 × 20 cm^3^ solid water phantom (CIRS, Norfolk, VA) and a CIRS thorax phantom (CIRS, Norfolk, VA). The solid water phantom was used to verify the necessity of modeling off-axis data. The model was also tested on a CIRS thorax phantom with 20 RT plans (10 IMRT and 10 VMAT), including 6 head and neck, 7 breast, and 7 rectum plans. The predicted TI is compared with the measured TI using a global gamma analysis of 3%/2 mm with a 10% threshold to verify the model's accuracy.

### Model sensitivity

A sensitivity test of our model to detect errors was performed by introducing several types of errors into the treatment plan and phantom setup. This was done using six fields (two head and neck, two breast, and two rectum), and the unperturbed predicted TI was compared to the perturbed measured TI using the global gamma analysis of 3%/3 mm (10% threshold). The perturbed plans were delivered to the thorax phantom, and the errors were introduced as follows:

- The number of monitor units for all field was increased by + 1% (e1), + 3% (e2), + 5% (e3), + 10% (e4), and − 5% (e5).
- The phantom was offset by 5 mm (e6), 10 mm (e7), and 20 mm (e8) laterally toward the right and by the same offsets in the anterior direction (e9–e11).
- The MLC leaves on all control points were opened by 5 mm (e12); both banks shifted in the same direction by 5 mm (e13); the leaves of bank B that were within the field were shifted by 5 mm (e14); the central four leaf-pairs on all control points were opened by 10 mm (e15).
- The gantry angles of the IMRT field were offsets of + 5° (e16) and + 10° (e17).

## Results

### Parameters

#### Kernel, linear function and horn correction map

The parameters of the kernel function in Eqs. (3), (4), and (5) are: $c$ = 0.262, $\sigma = 1.523$ cm, $a_{1} = 28.9707$, $a_{2} = 0.08528$, $a_{3} = 0.003675$, $b_{1} = 22.8806$ cm^−1^,$b_{2} = 10.27858$ cm^−1^, $b_{3} = 0.614335$ cm^−1^, $A = 2.67 \times 10^{5}$, and $B = - 168$.

**Fig. 4.**
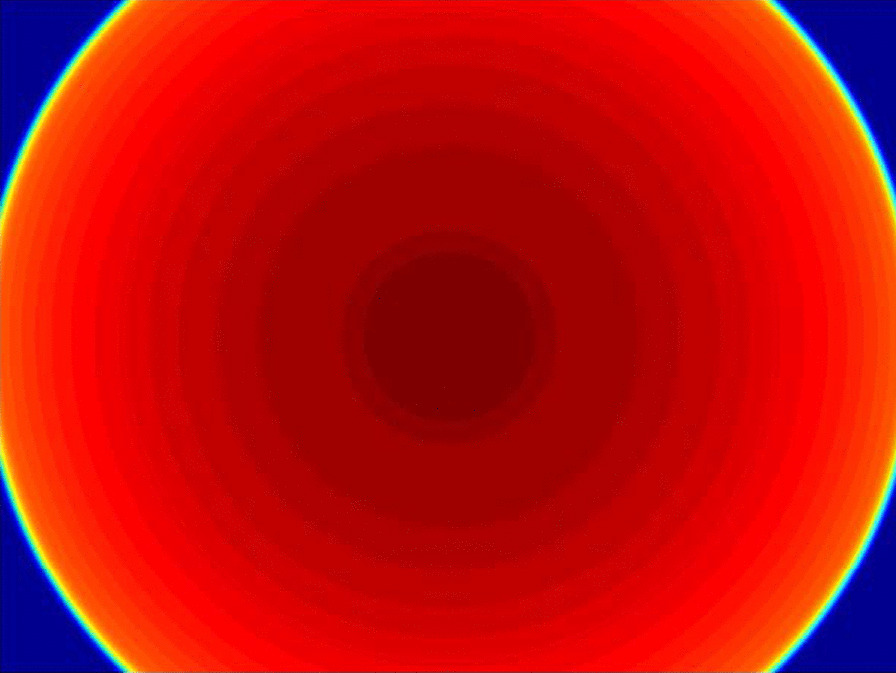
2D Horn correction map

**Fig. 5:**
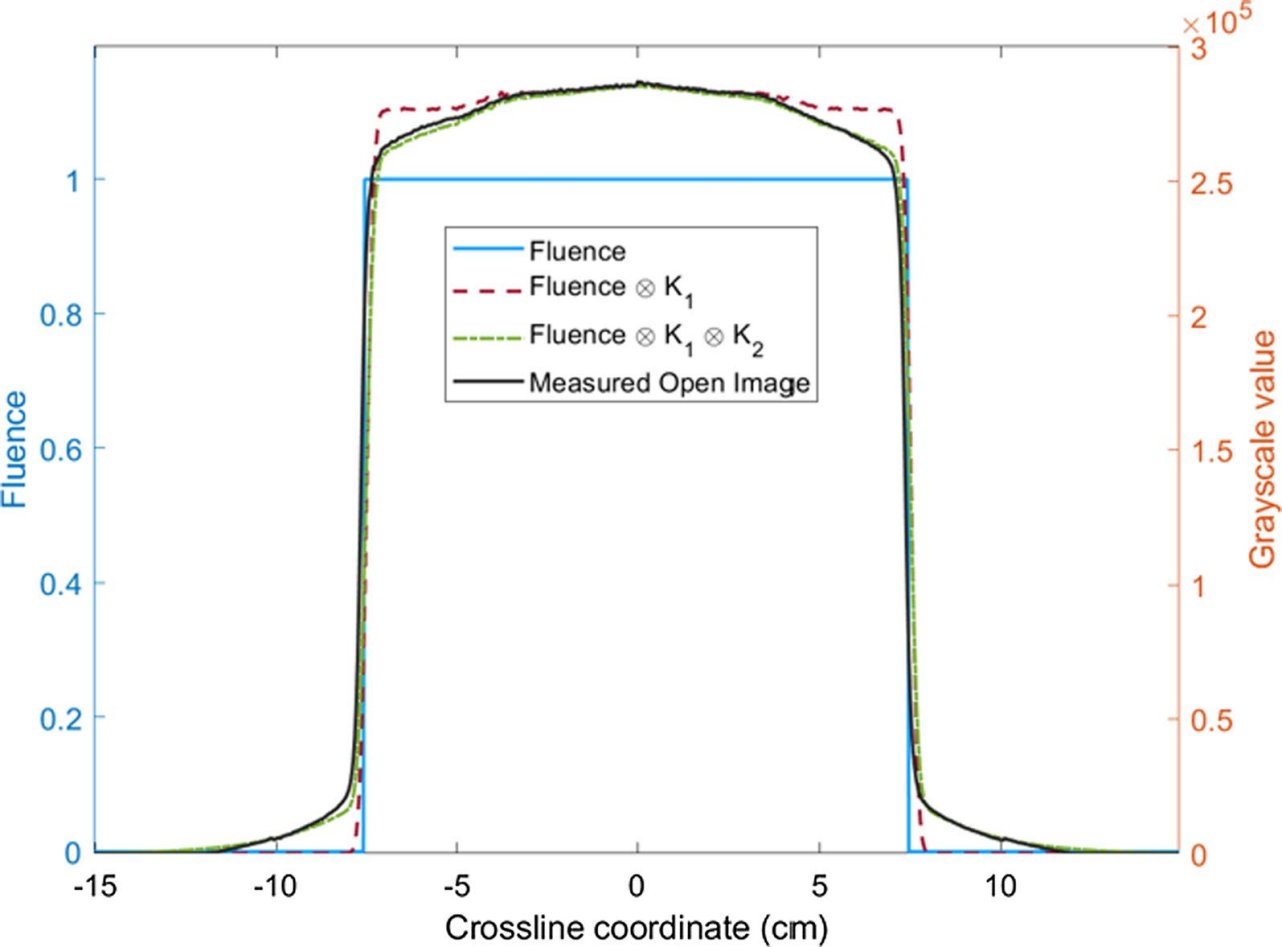
The crossline value of the fluence value (blue solid line) of 10 cm × 10 cm field, the open portal image calculated from the fluence map convolution with k1 (red dotted line), the open portal image calculated from the fluence map convolution with k1and k2 (green dotted line), and the measured open portal image (black solid line)

Figure 4 is the two-dimension horn correction map. The fluence map of a 10 × 10 cm^2^ field is converted into the open portal image after penumbra correction, response correction, and horn correction. Compared with the measured image, the absolute deviation ($100 \times \frac{{\left| {measured - predicted} \right|}}{measured}$) is < 1%, as shown in Fig. 5.

#### Global field output factor

**Fig. 6:**
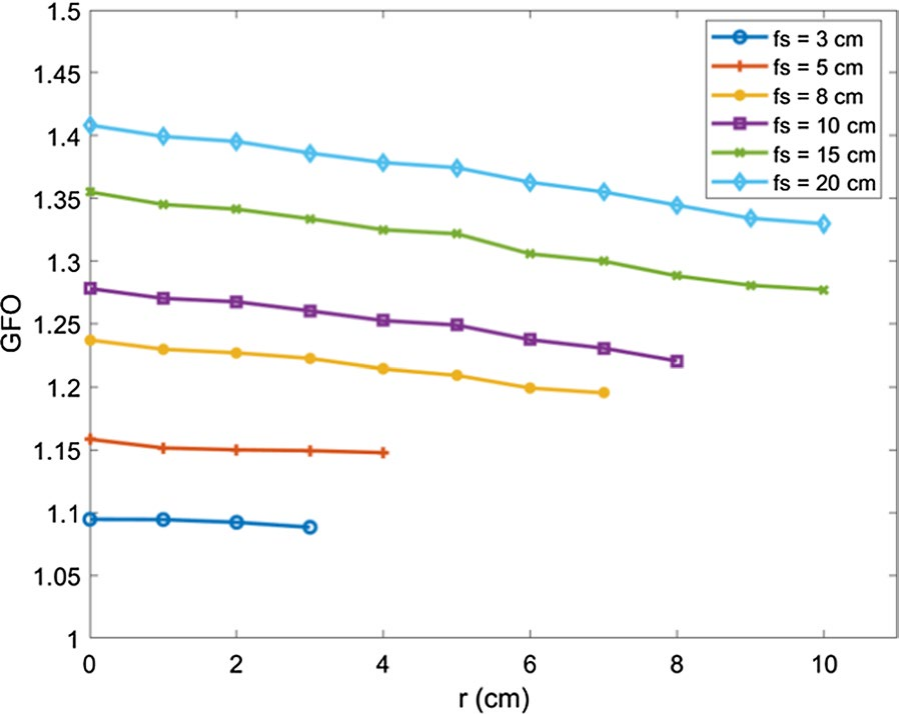
The global field output factor for different field sizes (3 cm, 5 cm, 8 cm, 10 cm, 15 cm, 20 cm) and various off-axis distances

Since the scatter value of the off-axis is different from that of the central axis, the GFO of the central axis and off-axis points are different. Therefore, the GFO with different off-axis distances are calculated separately in the open image. For the same field, with the increasing off-axis distance, the scatter value gradually decreases and the GFO value decreases. For the same off-axis distance, the scatter value increases with the increasing field size, so the GFO value increases. Figure 6 shows the GFO for different field sizes.

#### Attenuation coefficients

**Fig. 7:**
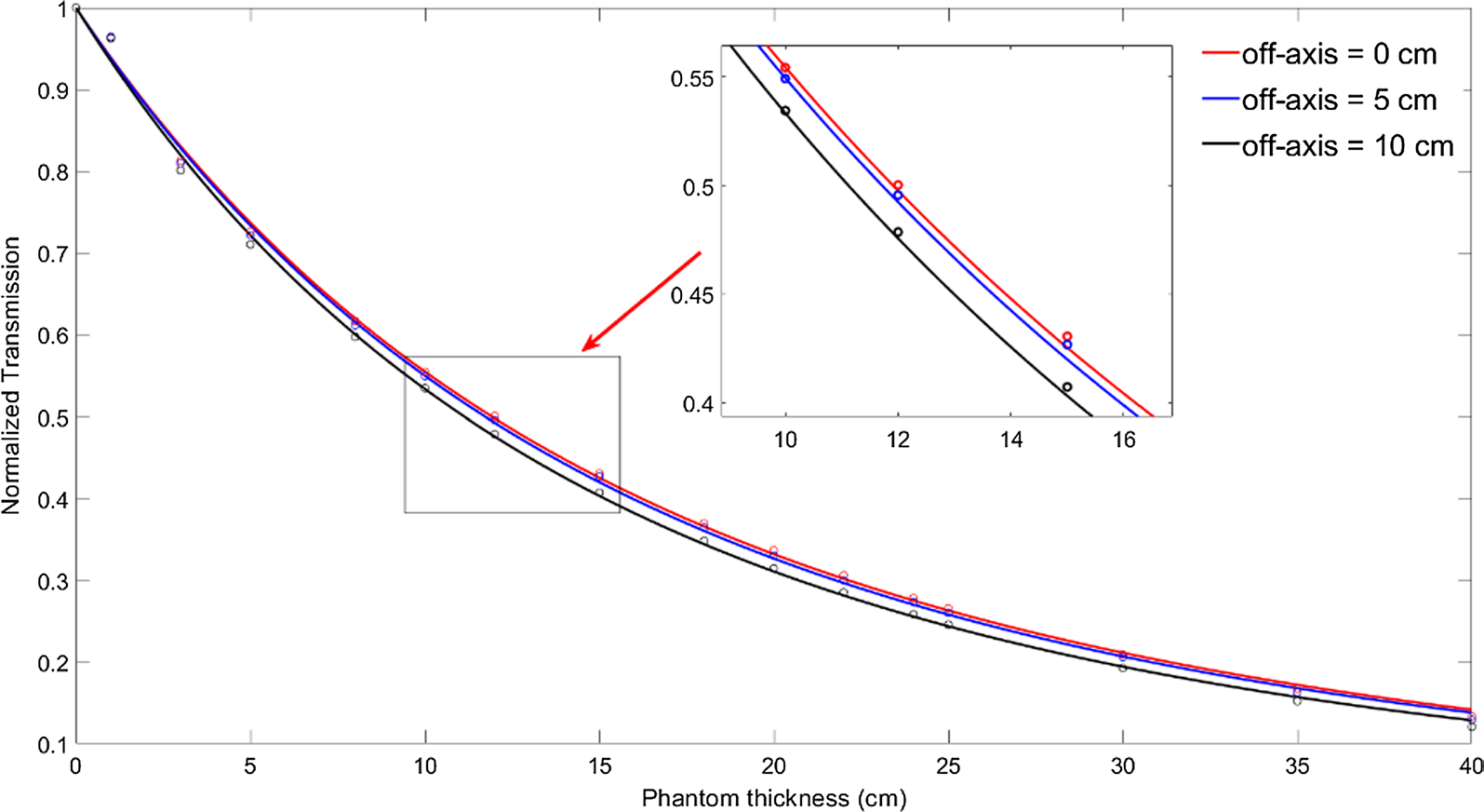
Transmission through solid water of varying thickness along the in-axis (red), off-axis distance is 5 cm (blue) and off-axis distance is 10 cm (black). The dots correspond to the measured data and the lines to the corresponding fitted curves using the parameters in Table 2 by Eq. (13)

In the process of X-rays penetrating the phantom, a softening effect will appear with increasing off-axis distance, so the attenuation coefficients of the central axis and off-axis are calculated separately. Table 2 shows the attenuation coefficients of the primary X-rays to solid water at different off-axis positions. Figure 7 shows the measured data and corresponding fitted curves using Eq. (13).

**Table 2:** Attenuation coefficients of different off-axis distances

r/cm	0	1	2	3	4	5	6	7	8	9	10
$a(r)$	0.0575	0.0596	0.0598	0.0600	0.0601	0.00606	0.0614	0.0617	0.0628	0.0635	0.0640
$b(r)$	0.003642	0.005063	0.005039	0.004948	0.005014	0.005135	0.00536	0.005288	0.005645	0.005791	0.005898

#### Scatter to primary ratio

**Fig. 8:**
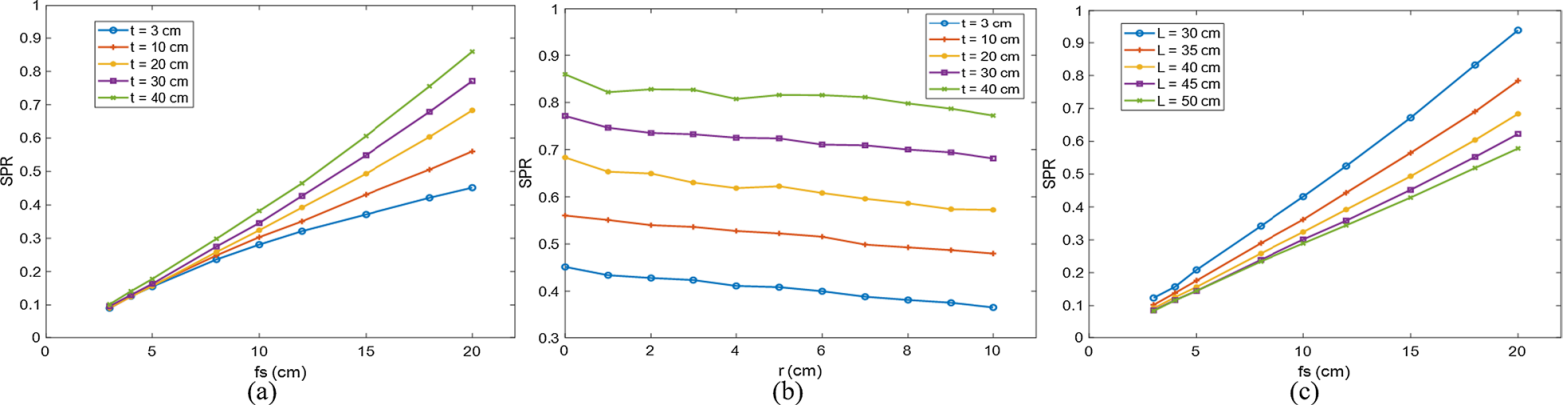
The SPR of the EPID under different conditions. **a** Shows the central axis SPRs of different thicknesses (3, 10, 20, 30, and 40 cm) and different field sizes (3–20 cm). **b** Shows the off-axis SPRs of different thicknesses and different off-axis distances (0–10 cm) when the field size is 20 × 20 cm^2^. **c** Shows the central axis SPRs of different exit gaps and different field sizes (3–20 cm) when the thickness is 20 cm

The scatter value in the EPID TI is related to the field size, phantom thickness, off-axis distance, and exit air gap, so we modeled the SPR of the data in Table 1. For SPR modeling, due to the impact of X-rays softening and hardening effects, the primary fluence and the scatter fluence of the field's central axis were not the same as those of the off-axis points. As the field size and phantom thickness increase, the scatter value increases, resulting in an increase in SPR (Fig. 8a). When the phantom thickness is constant, with increasing off-axis distance, the scatter value decreases, making the SPR decrease overall (Fig. 8b). As the exit gap increases, a part of the low-energy scattered rays disappears in the air, causing the SPR to decrease (Fig. 8c). Therefore, in addition to considering the influence of different field sizes, different phantom thicknesses, and various air gaps, different off-axis effects were also considered.

### Validation

#### Calculation of the off-axis parameters

**Fig. 9:**
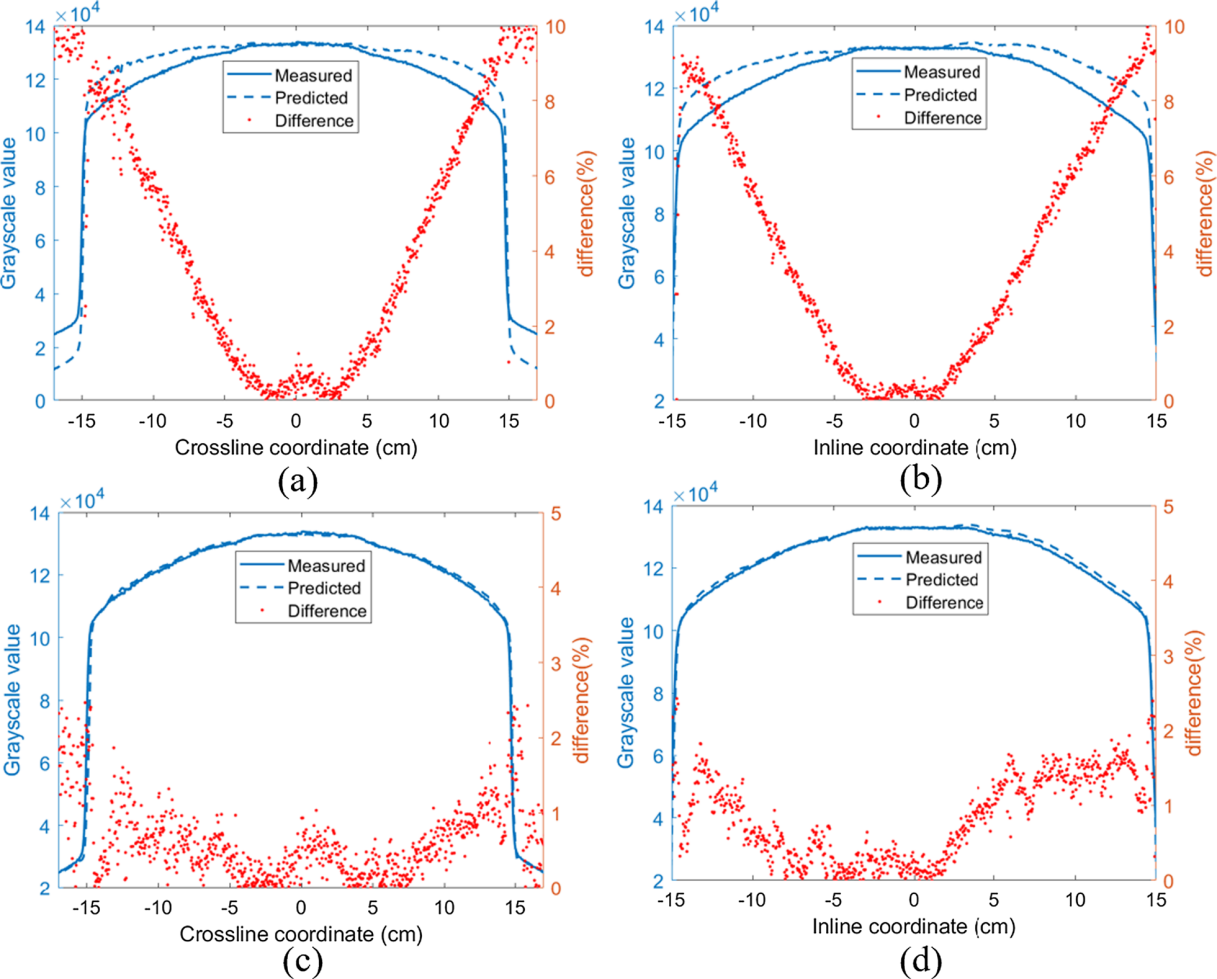
The transmission image calculated (blue dotted line) from the fluence map, the measured image (blue solid line), and the relative error (red point). **a,** **b** Are the crossline and inline directions calculated using the in-axis parameters, respectively. **c**, **d** Are the crossline and inline directions calculated using the off-axis parameters, respectively

To verify the necessity of modeling off-axis data, the fluence of the 20 × 20 cm^2^ field (at the EPID level, 30 × 30 cm^2^) was used to predict the TI using off-axis parameters and using only central axis parameters, as shown in Fig. 9. The phantom used was 20 cm thick solid water, and the gantry angle was 0.

When the prediction model uses the parameters (global field output factor, attenuation coefficients and scatter to primary ratio) of the central axis to calculate the TI, compared with the measured TI, with increases in the off-axis distance, the error of the predicted image relative to the measured TI increases gradually. The error ($100 \times \frac{{\left| {measured - predicted} \right|}}{measured}$) is approximately 10% at the edge of the field. In contrast, the error is approximately 2% when the off-axis GFO, the off-axis attenuation coefficient, and the off-axis SPR are used to predict the TI.

#### IMRT and VMAT fields

**Fig. 10:**
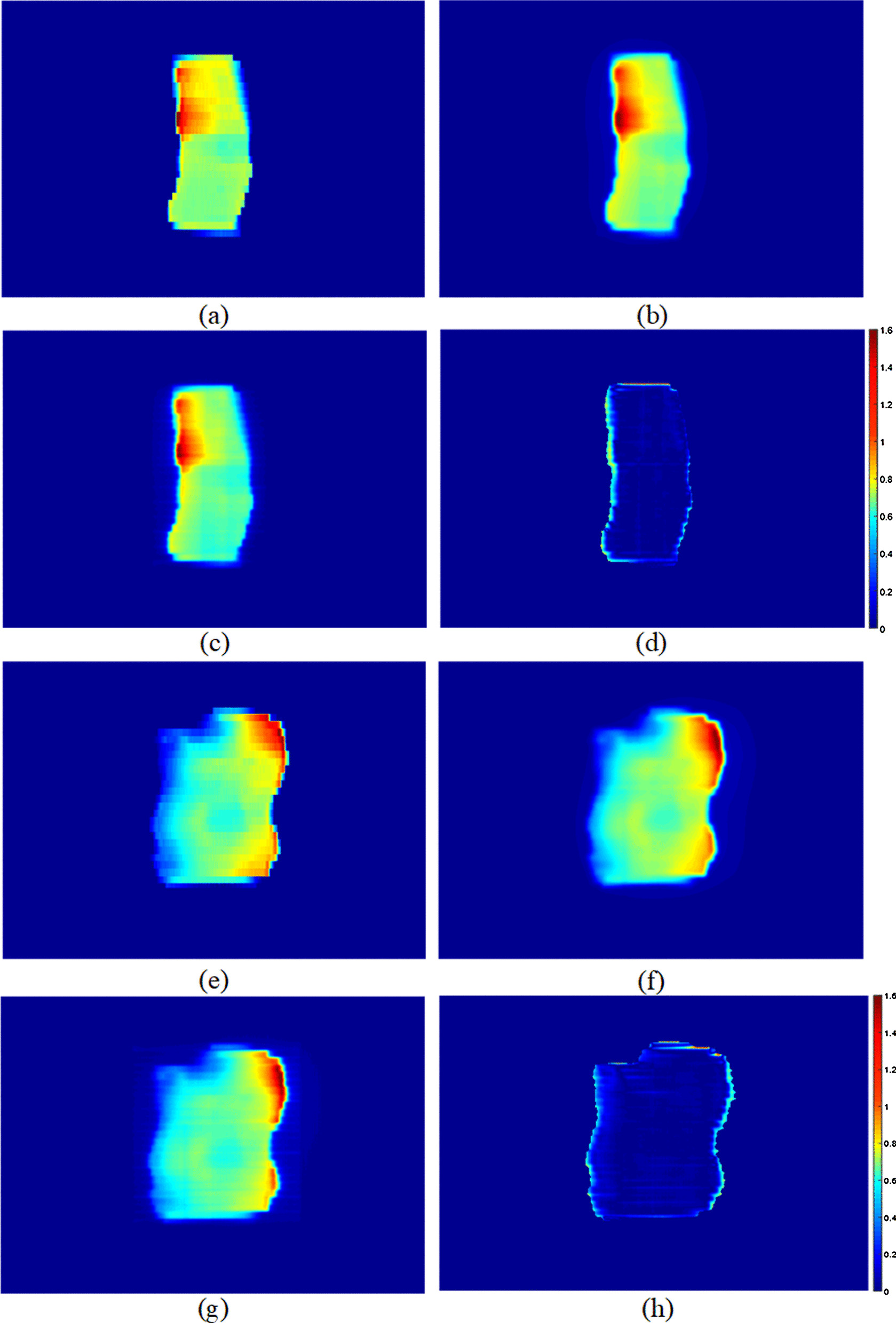
Two field of IMRT plan. **a**, **e** Are the fluence map exported from the RT-plan, **b**, **f** are the predicted transmission images, **c**, **g** are the measured transmission image, and **d**, **h** are the gamma comparisons between the measured and predicted images

**Fig. 11:**
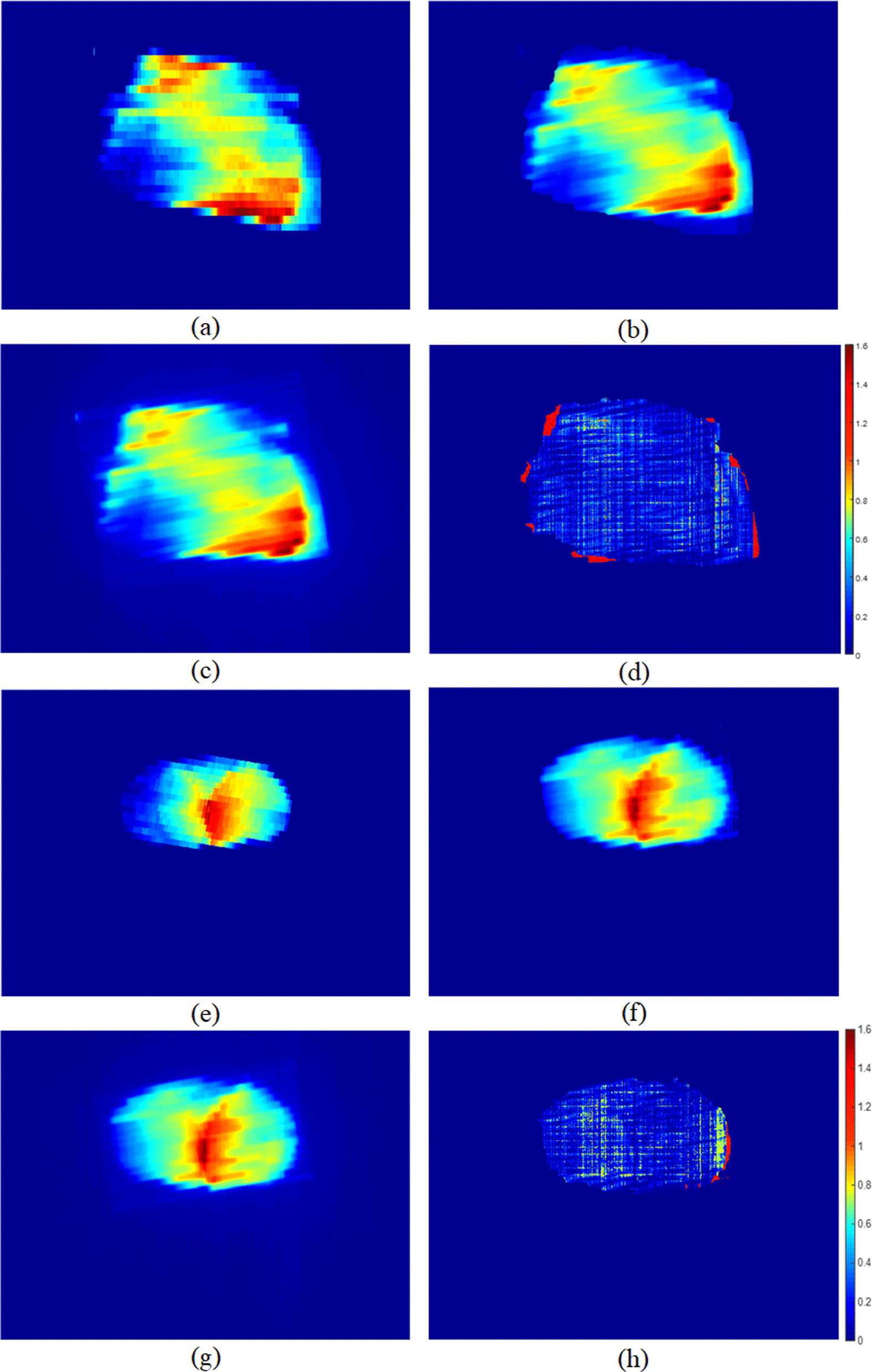
Two sample VMAT fields: **a**, **e** are the fluence map extracted from the RT plan, **b**, **f** are the predicted transmission images, **c**, **g** are the measured transmission images, and **d**, **h** are the gamma comparisons between the measured and predicted images

For the IMRT field and VMAT fields, the gamma index evaluation of the predicted TI compared to the measured image. The pass rates for IMRT and VMAT plans were > 97.2% and 94.5% (3%/2 mm, threshold 10%, global) respectively, and the average gamma index values were 0.28 and 0.49. Figures 10 and 11 show the results of two IMRT fields and two VMAT fields.

#### Model sensitivity

The gamma indices for the unperturbed predicted TI in relation to the perturbed measured TI are given in Table 3. It is clear that monitor unit errors have a larger impact on the distribution in the TI, and the increase in MU errors leads to a decrease in the pass rate of gamma criteria. When the MU error is greater than 5%, the gamma pass rate is less than 82%. The reported results from Table 3 show that the phantom offset in one direction does not result in a significant reduction in the gamma pass rate. The results of MLC errors described in Table 3 demonstrate that our model is sensitive to a range of MLC errors, and different types of MLC errors lead to a significant reduction in the gamma pass rate. For the gantry angle error of the IMRT field, when the gantry angle is offset by 5°, the result does not decrease significantly, and when the gantry angle is offset by 10°, the pass rate is less than 90%. As a result, our model is sensitive to the monitor unit error and MLC error, and less sensitive to the setup error and gantry angle error.

**Table 3:** Results of the sensitivity analysis

Plan error type	H&N 1 (IMRT)	H&N 2 (IMRT)	Breast 1 (IMRT)	Breast 2 (VMAT)	Rectum 1 (VMAT)	Rectum 2 (VMAT)
MU increased by 1% (e1)	97.6	98.2	98.4	97.5	97.3	97.8
MU increased by 3% (e2)	95.7	96.4	93.4	91.5	89.7	87.3
MU increased by 5% (e3)	76.4	81.2	79.3	72.7	69.8	75.9
MU increased by 10% (e4)	62.5	58.4	69.4	52.4	57.6	48.7
MU decreased by 5% (e5)	81.5	77.3	68.4	69.1	74.1	73.7
Offset by 5 mm toward right (e6)	98.2	97.6	98.2	96.5	94.8	96.1
Offset by 10 mm toward right (e7)	97.5	98.1	99.1	96.3	98.3	95.3
Offset by 20 mm toward right (e8)	93.4	92.5	94.7	91.3	92.4	94.8
Offset by 5 mm toward anterior (e9)	98.1	100	99.5	98.8	98.4	97.1
Offset by 10 mm toward anterior (e10)	97.9	98.8	97.8	96.7	95.2	94.9
Offset by 20 mm toward anterior (e11)	98.5	99.2	98.5	97.1	98.3	96.4
MLC leaves opened by 5 mm (e12)	48.2	54.5	39.1	29.8	37.2	19.7
MLC leaves shifted in same direction by 5 mm (e13)	79.8	68.5	59.4	64.4	56.9	70.1
MLC leaves of bank B within the field shifted by 5 mm (e14)	56.4	49.7	43.6	38.4	27.9	40.6
MLC leaves of central four leaf-pairs opened by 10 mm (e15)	76.9	80.7	84.1	79.3	73.4	69.5
Gantry angle offset by + 5	96.3	94.2	94.7			
Gantry angle offset by + 10	82.6	86.9	79.4			

## Discussion

In this study, we used the fluence map to predict the TI. The predicted TI was compared with the measured TI for in vivo treatment verification. All parameters used in the model are calculated using the commissioning EPID measurement data.

Li et al. [28] demonstrated that different regions use the same kernel, resulting in discrepancies between computed and measured images. In our model, the global field output factor, attenuation coefficients, and scatter to primary ratio in different regions are calculated.

As mentioned in the literature review, Pasma et al. [36] established a scatter model using the central axis EPID measurement data and an ionization chamber. Since the scattered values of the central axis and off-axis points are different, calculation errors may be introduced when only the measurement data of the central axis are used (Fig. 9a, b). Therefore, it is necessary to model the three parameters at the off-axis point, which can reduce the calculation error of the predicted algorithm (Fig. 9c, d). Due to the overresponse of low energy rays at the edge of the field [2], the error at the field edge is relatively large, but the overall gamma pass rates (3%/2 mm, threshold 10%, global) are > 97.2% and 94.5% for IMRT and VMAT, respectively. Therefore, it can be used for in vivo treatment verification in the clinic.

In contrast to other forward dosimetry verification solutions, van Elmpt et al. [8] also only used EPID measurement data for modeling. They acquired EPID open images without patients before treatment and then used open images to predict the EPID TIs. The drawback of this method is that it may lead to incorrect judgment of the verification results. For example, if there is a machine error in the acquisition of the open image, but there is no machine error in the treatment process, the verification result will be wrongly judged. Similarly, if the machine delivers an error, the error will be present in the open image, the predicted image, and the measured image. When comparing the predicted image with the measured image, the error may be undetected. In addition, this method requires repeated execution of the treatment plan before treatment, which is time-consuming work. The reported results from Table 3 show that our model is less sensitive to the phantom setup errors and gantry angle error. Theoretically, these errors will lead to the inconsistency between the predicted TI and the measured TI, as the sensitivity of the model to those errors mainly depends on the equivalent thickness of the ray passing through the phantom. However, for the CIRS thorax phantom, the small deviation of the position has no obvious effect on the change in equivalent thickness and little influence on the TI. These results agreed with the results reported by Najem et al. [37]. For the same reason, the result does not change significantly when the gantry angle error is 5°.

van Zijtveld et al. [9] converted the idealized fluence into an EPID open image through head scatter correction (long-range kernel) and penumbra correction (short-range kernel) and calculated the attenuation coefficient with an infinitely small field. The open image was multiplied by the attenuation coefficient to calculate the primary ray of the TI. The open image contains the primary ray and the scatter ray from the Linac head and the internal EPID. However, when the field is infinitely small, only the primary ray and no other scatter ray are generated, so this method only calculates the attenuation coefficient of the primary ray, which is not suitable for calculating the attenuation of the scatter ray. It is not accurate to calculate the primary ray in EPID TI by multiplying the open image with the attenuation coefficient of the primary ray. Berry et al. [38] converted the open image predicted by Varian Portal Dosimetry into the TI, but this method is only applicable to the fixed air gap (35 cm) between the phantom exit and EPID. The EPID needs to be moved for each beam to ensure that the air gap is constant, which is difficult to achieve in VMAT. Najem et al. [37] improved Berry's method, which can be used in any exit gap. In this method, the open image was calculated by an empirical attenuation correction factor $T(t,fs)$ to obtain the TI. Similarly, the open image contains the primary ray and the scatter ray. The primary ray follows the exponential attenuation law, and the scatter model is more complicated, which is related to the field size, the thickness of the phantom, and the air gap. However, their attenuation correction factor does not consider the effect of the exit gap. Finally, they corrected it with an air gap factor. When calculating the air gap factor, the acquired image contains the primary ray and the scatter ray, but the primary ray is not affected by the air gap, and the expression of the air gap factor is not a linear equation, which cannot eliminate the influence of the primary ray.

In our algorithm, the fluence map is extracted from the RT plan to calculate the EPID TI directly, so there is no need to perform pretreatment acquisition of the open portal image of the RT plan. The primary ray and the scatter ray were modeled, the scatter ray in the open image was removed and the primary ray was calculated by superposition of the scatter ray after attenuation correction to obtain the predicted TI, which is more accurate for the modeling of the primary ray and the scatter ray. Chytyk-Praznik et al. [11, 14] and Woodruff et al. [20] used the MC method to calculate the scatter kernel of the EPID. This process requires in-depth knowledge and detailed structures of Linac and EPID, which is challenging for clinical physicists. In addition, Monte Carlo requires a long calculation time while obtaining high accuracy, which is difficult to widely use in clinical treatment. In our model, all parameters were calculated using EPID measurement data (central axis and off-axis), and no other dose measurement tools were necessary. Furthermore, there is no need to use the Monte Carlo method or a convolution method to calculate the scatter value of the EPID; the scatter values of different off-axis points were modeled separately, so the calculation process is accurate and simple.

## Conclusion

We have developed an accurate and straightforward EPID-based quality assurance protocol for in vivo treatment verification in RT delivery. The model uses the fluence map extracted from the RT plan to predict the TI, the primary and scatter rays are modeled, and the predicted TI is compared with the measured TI for in vivo treatment verification. The parameters (global field output factor, attenuation coefficients and scatter to primary ratio) used in the model are calculated separately for central axis and off-axis points using EPID measurement data. Our model avoids using convolution or iteration methods to calculate the scatter value in TI, simplifies the calculation process, and improves the calculation accuracy of off-axis points. The gamma pass rate compared with the calculated image and the measured image is above 94% in this study. Thus, the proposed method can be used for in vivo treatment verification in the clinic.

## Data Availability

The datasets during the current study are not publicly available due to some research that has not been completed, but is available from the corresponding author on reasonable request.

## References

[1] Low DA, Moran JM, Dempsey JF, Dong L, Oldham M (2011). Dosimetry tools and techniques for IMRT. Med Phys.

[2] Greer PB (2005). Correction of pixel sensitivity variation and off-axis response for amorphous silicon EPID dosimetry. Med Phys.

[3] van Elmpt W, Nijsten S, Petit S, Mijnheer B, Lambin P, Dekker A (2009). 3D in vivo dosimetry using megavoltage cone-beam CT and EPID dosimetry. Int J Radiat Oncol Biol Phys.

[4] McCowan PM, Van Uytven E, Van Beek T, Asuni G, McCurdy BM (2015). An in vivo dose verification method for SBRT-VMAT delivery using the EPID. Med Phys.

[5] Bailey DW, Kumaraswamy L, Bakhtiari M, Malhotra HK, Podgorsak MB (2012). EPID dosimetry for pretreatment quality assurance with two commercial systems. J Appl Clin Med Phys.

[6] Woodruff HC, Fuangrod T, Rowshanfarzad P, McCurdy BM, Greer PB (2013). Gantry-angle resolved VMAT pretreatment verification using EPID image prediction. Med Phys.

[7] Camilleri J, Mazurier J, Franck D, Dudouet P, Latorzeff I, Franceries X (2016). 2D EPID dose calibration for pretreatment quality control of conformal and IMRT fields: a simple and fast convolution approach. Phys Med.

[8] van Elmpt WJ, Nijsten SM, Mijnheer BJ, Minken AW (2005). Experimental verification of a portal dose prediction model. Med Phys.

[9] van Zijtveld M, Dirkx M, Breuers M, de Boer H, Heijmen B (2009). Portal dose image prediction for in vivo treatment verification completely based on EPID measurements. Med Phys.

[10] Wendling M, McDermott LN, Mans A, Sonke JJ, van Herk M, Mijnheer BJ (2009). A simple backprojection algorithm for 3D in vivo EPID dosimetry of IMRT treatments. Med Phys.

[11] Chytyk K, McCurdy BM (2009). Comprehensive fluence model for absolute portal dose image prediction. Med Phys.

[12] Martinez Ortega J, Pinto Monedero M, Gomez Gonzalez N, Tolani NB, Castro Tejero P, Castanedo Alvarez M, Nunez Martin L, Sanchez Montero R (2018). A collapsed-cone based transit EPID dosimetry method. Phys Med.

[13] Wendling M, McDermott LN, Mans A, Olaciregui-Ruiz I, Pecharroman-Gallego R, Sonke JJ, Stroom J, van Herk M, Mijnheer BJ (2012). In aqua vivo EPID dosimetry. Med Phys.

[14] Chytyk-Praznik K, VanUytven E, vanBeek TA, Greer PB, McCurdy BM (2013). Model-based prediction of portal dose images during patient treatment. Med Phys.

[15] Van Uytven E, Van Beek T, McCowan PM, Chytyk-Praznik K, Greer PB, McCurdy BM (2015). Validation of a method for in vivo 3D dose reconstruction for IMRT and VMAT treatments using on-treatment EPID images and a model-based forward-calculation algorithm. Med Phys.

[16] Fuangrod T, Greer PB, Woodruff HC, Simpson J, Bhatia S, Zwan B, vanBeek TA, McCurdy BM, Middleton RH (2016). Investigation of a real-time EPID-based patient dose monitoring safety system using site-specific control limits. Radiat Oncol.

[17] van Elmpt W, McDermott L, Nijsten S, Wendling M, Lambin P, Mijnheer B (2008). A literature review of electronic portal imaging for radiotherapy dosimetry. Radiother Oncol.

[18] Margalit DN, Chen YH, Catalano PJ, Heckman K, Vivenzio T, Nissen K, Wolfsberger LD, Cormack RA, Mauch P, Ng AK (2011). Technological advancements and error rates in radiation therapy delivery. Int J Radiat Oncol Biol Phys.

[19] Mans A, Remeijer P, Olaciregui-Ruiz I, Wendling M, Sonke JJ, Mijnheer B, van Herk M, Stroom JC (2010). 3D Dosimetric verification of volumetric-modulated arc therapy by portal dosimetry. Radiother Oncol.

[20] Woodruff HC, Fuangrod T, Van Uytven E, McCurdy BM, van Beek T, Bhatia S, Greer PB (2015). First experience with real-time EPID-based delivery verification during IMRT and VMAT sessions. Int J Radiat Oncol Biol Phys.

[21] Fuangrod T, Woodruff HC, van Uytven E, McCurdy BM, Kuncic Z, O'Connor DJ, Greer PB (2013). A system for EPID-based real-time treatment delivery verification during dynamic IMRT treatment. Med Phys.

[22] Hansen VN, Swindell W, Evans PM (1997). Extraction of primary signal from EPIDs using only forward convolution. Med Phys.

[23] McCurdy BM, Pistorius S (2000). Photon scatter in portal images: physical characteristics of pencil beam kernels generated using the EGS Monte Carlo code. Med Phys.

[24] Wang S, Gardner JK, Gordon JJ, Li W, Clews L, Greer PB, Siebers JV (2009). Monte Carlo-based adaptive EPID dose kernel accounting for different field size responses of imagers. Med Phys.

[25] Spies L, Bortfeld T (2001). Analytical scatter kernels for portal imaging at 6 MV. Med Phys.

[26] Warkentin B, Steciw S, Rathee S, Fallone BG (2003). Dosimetric IMRT verification with a flat-panel EPID. Med Phys.

[27] Steciw S, Warkentin B, Rathee S, Fallone BG (2005). Three-dimensional IMRT verification with a flat-panel EPID. Med Phys.

[28] Li W, Siebers JV, Moore JA (2006). Using fluence separation to account for energy spectra dependence in computing dosimetric a-Si EPID images for IMRT fields. Med Phys.

[29] Passarge M, Fix MK, Manser P, Stampanoni MF, Siebers JV (2017). A Swiss cheese error detection method for real-time EPID-based quality assurance and error prevention. Med Phys.

[30] Alves VGL, Ahmed M, Aliotta E, Choi W, Siebers JV (2021). An error detection method for real-time EPID-based treatment delivery quality assurance. Med Phys.

[31] Van Esch A, Depuydt T, Huyskens DP (2004). The use of an aSi-based EPID for routine absolute dosimetric pre-treatment verification of dynamic IMRT fields. Radiother Oncol.

[32] Winkler P, Hefner A, Georg D (2007). Implementation and validation of portal dosimetry with an amorphous silicon EPID in the energy range from 6 to 25 MV. Phys Med Biol.

[33] Ahmad M, Nourzadeh H, Siebers J (2021). A regression-based approach to compute the pixels sensitivity map of linear accelerator portal imaging devices. Med Phys.

[34] Siddon RL (1985). Fast calculation of the exact radiological path for a three-dimensional CT array. Med Phys.

[35] Pecharroman-Gallego R, Mans A, Sonke JJ, Stroom JC, Olaciregui-Ruiz I, van Herk M, Mijnheer BJ (2011). Simplifying EPID dosimetry for IMRT treatment verification. Med Phys.

[36] Pasma KL, Heijmen BJ, Kroonwijk M, Visser AG (1998). Portal dose image (PDI) prediction for dosimetric treatment verification in radiotherapy. I. An algorithm for open beams. Med Phys.

[37] Najem MA, Tedder M, King D, Bernstein D, Trouncer R, Meehan C, Bidmead AM (2018). In-vivo EPID dosimetry for IMRT and VMAT based on through-air predicted portal dose algorithm. Phys Med.

[38] Berry SL, Sheu RD, Polvorosa CS, Wuu CS (2012). Implementation of EPID transit dosimetry based on a through-air dosimetry algorithm. Med Phys.

